# Market Fashioning

**DOI:** 10.1007/s11186-020-09379-0

**Published:** 2020-02-05

**Authors:** Patrik Aspers, Petter Bengtsson, Alexander Dobeson

**Affiliations:** 1 Seminar for Sociology, University of St. Gallen, SfS-HSG Müller-Friedberg-Strasse 6/8, 9000 St. Gallen, Switzerland; 2 Department of Sociology, Stockholm University, 10691 Stockholm, Sweden; 3 Department of Sociology, Uppsala University, Box 624, 751 26, Uppsala, Sweden

**Keywords:** Economic sociology, Exchange, Field, Marketplace, Mutual adjustment, Organization

## Abstract

How do markets come about? This article offers a first systematic analysis of three different ideal types of market fashioning: mutual adjustment, organization, and fields. Although aspects of these are identifiable in most empirical markets, these three ideal types provide analytic tools for students of real markets and marketplaces. After going through this comprehensive literature, it is argued that mutual adjustment, which refers to non-planned processes, is affinity with markets in which products are differentiated, for example, producer markets. Organization refers to process driven by attempts to decide for others and shows affinity with markets for standardized and homogenous products, for example, stock exchanges. Organization also accounts for the making of marketplaces. The broader notion of fields does not refer to any specific process, but accounts for the context of market fashioning and its respective power struggles.

Markets occupy a central role in contemporary social life. Empirically existing markets have been studied and theorized, and the sociology of markets has reached a mature stage, as existing reviews indicate (e.g., Aspers 2011; Fligstein and Dauter 2007; Lie 1997; Swedberg 2005). How markets come about has, however, received little attention, both empirically and theoretically (Fligstein 2001a, p. 14).

It is against the backdrop of marketization, economization, and other contested concomitant social processes, and that we know little of how they come about, that a deeper understanding of market fashioning is needed, we argue. By “market fashioning” we mean the way markets and marketplaces result from human undertakings. Given that the conditions of a market, defined below, are met, we may ask how a specific new market and marketplaces are fashioned. The etymology of fashion is traceable to the Latin word *factionem*, which refers to the making and doing something together. “Fashioning,” hence, denotes the social activity of making something and is broad enough to cover both emergence and organization of markets, two different ideas that are prevalent in the existing literature. Emergence has a clear reference to a natural, unintentional, and non-planned process bound by law (Sawyer 2001). Organization refers to the intentional attempt to make something by deciding for others (Luhmann 2000). Moreover, change of existing markets and fashioning of new markets are different but related issues. There is more written on change of existing markets than on how new markets come about (Geiger and Gross 2018). “Changes” in existing markets, however, occur more or less regularly, while the process of how new markets come about is theoretically more distinct. To illuminate this surprisingly neglected issue in the relatively large literature on markets, a systematic review of different forms of market fashioning is at the heart of this text, which is the first of its kind.

Our method is to conduct a meta-analysis of the underlying ideal types of market fashioning in the existing literature on markets. The natural starting point is the historical literature on markets, though the first “markets” where marketplaces, some of which preceded cities, some of which were licensed to cities by a king or a bishop (Britnell 2009, pp. 48-54). The question of what we call market fashioning has been addressed in several studies (e.g., van Bavel 2016), but the theoretical outcome is relatively meager. This has two reasons, partly due to the way the questions have been posed and the lack of theories suitable for the job, partly because the problem of having access to detailed enough empirical material to perform the type of analysis required. Of course, our survey cannot be exhaustive and we limit our choice to a few exemplary approaches of market fashioning. We zoom in on sociology and economics and identify three ideal typical (Weber 1949, pp. 42-45) processes of market fashioning in the existing research. According to one ideal type, markets result from actors’ mutual adjustment, which means that markets are unintended consequences of trade. This is the ideal type that many economists, especially “Austrians,” adhere to, but some sociologists also use it as point of departure, for example, Harrison White in his theory of producer markets. We then observe that there is affinity with the process of mutual adjustment, and the form of market, characterized by differentiation of products, producers, and consumers. A second ideal type refers to attempts to organize markets, which nonetheless does not mean that the result is according to anyone’s plan. This ideal type guides organization scholars and the performativity camp, but also some economists, such as Alfred Marshall and Leon Walras. This process shows affinity with markets in which the products are more or less standardized or homogenous; such as exchanges, for metal, emission rights, or stocks. Organization is also the process that shows affinity with how marketplaces are fashioned. The third ideal type, “fields,” is somewhat different than the other two. There is no affinity with a specific market type, since this ideal type of market fashioning is not focusing on the actual market, what is exchanged and the like, but the context and its respective political power struggles. Seen in the light of fields, markets are often outcomes of struggles or cooperation between social movements/actors in an existing field to create something new. This ideal type of market fashioning is typical of field theorists, such as Pierre Bourdieu and Neil Fligstein.

We are studying the literature on empirical markets and their fashioning, not fashioning of society at large, which has been the focus of general sociology (Sztompka 1993). Thereby we offer a stepping-stone to the much needed theorizing on the often complex and multi-faceted process of market fashioning. We conclude with some intriguing questions about the making of markets and marketplaces for future research.

## Marketplaces, markets, and the market society

Scientific work on market fashioning can be concerned with both individual markets and marketplaces, or as Sombart calls them, abstract and concrete markets (Sombart 1927, Vol. II, p. 528). The term “marketplace”, as we define it, denotes a location, whether physical (such as a weekly occurring event for trading in the town square), or virtual (in the form of a platform such as eBay), where a variety of products are traded by buyers and sellers. Historically, it is correct to say that markets grew out of marketplaces (Braudel 1992; Jevons 1871, p. 84). A marketplace can be composed of or, in other terms, harbor several different markets. A stock exchange or eBay are marketplaces in which individual stocks and separate objects, such as records, books, or old miniature trains, are traded in their respective markets; i.e., they comprise different *items* of trade, different prices, and sometimes different ways of setting prices/establishing what is valuable.

We use a minimum market definition to frame the object of study. A market is here defined as a social structure for the exchange of owners’ rights, in which offers are evaluated and priced, and in which individuals or organizations compete with one another via offers (cf. Aspers 2011, p. 4). “Rights” is defined broadly enough to include all forms of “rights” to a service or other form of agreement on various types of exchanges including, for example, derivatives. A market is ordered, but how this occurs is a question addressed by both sociologists and economists (Beckert 2002; Nelson and Winter 2002). Any market structure is primarily constituted by two roles: that of buyer and seller, each with their own goal, “to sell at a high price” or to “buy at a low price” (Geertz 1992, p. 226); this end may be achieved in markets but also under monopoly or exchange. The opposing interest is typical of trade and not restricted to markets. In a market, one side, either the buying side or the selling side, must be composed of at least two actors, so that the offers can be evaluated in relation to each other by the other side, which means that there can be competition (Simmel 1923), often in terms of price, but also in terms of other “variables” (cf. Chamberlin 1953, p. 110), such as quality of what is traded or return policies. A market has prerequisites: (i) it is about something, (ii) there is a culture of how things are done, a notion that includes practices and institutions regarding how things are done in the market, and (iii) there is a way of setting prices. If one is to speak of a new market, these defining traits and prerequisites must be fashioned. Our question, hence, is concerned with how a market or marketplace comes about or changes, not what a marketplace or market is.

In all markets, prices and exchange results from mutual adjustment by a multitude of interactions and decisions—to buy or not to buy and to sell or not to sell—by those who trade in the market. Most, if not all, would agree that there are institutional conditions providing the bedrock of all organized markets (von Hayek 1973, pp. 46-48), yet still the market-fashioning process is unclear.

Our discussion of the three ideal types of how markets and marketplaces are fashioned that we have identified is structured in a similar way: first, we discuss the main ideas and the underlying assumptions, then we go into the details of the identified approach. Next, we present some empirical work to show market fashioning “in practice.” Before concluding, we briefly compare and discuss the three ideal types, and present ideas for future research.

## Mutual adjustment

Prices in virtually all existing markets are set in processes of actors who mutually adjust to one another. Our focus, however, is on how actors mutually adjust to one another’s behavior, which leads to the fashioning of markets and marketplaces. The core idea of the ideal type mutual adjustment—that actors adjust to one another in an ongoing interaction and observation of one another that leads to order (Lindblom 2001)—has been taken for granted as the starting point of market fashioning by proponents as well as opponents of market solutions. Many of those who problematize the market and often favor state intervention assume that markets result from mutual adjustment (Boyer 1990, 2005; Streeck 2005). Polanyi, perhaps more than any other thinker, has stated that the capitalistic economic system is “capable of creating a specific institution, namely the market” (Polanyi 2001, p. 60). A market, according to this view, is a grown and unintended order resulting from many individual decisions; there is no overall plan, but the result may very well be an order.

Mutual adjustment is sometimes called “spontaneous (order)” (von Hayek 1973, pp. 39-52) or “self-regulation,” which means that “all productions for sale on the market and incomes derive from such sales … [and that] [n]othing must be allowed to inhibit the formation of markets” (Polanyi 2001, p. 72). Sombart (1927, Vol. II, p. 530) calls this a “natural” (*natürlichen*) process. These notions refer to ancient ideas of evolution to account for how markets come about. In our view, the term is neutral in relation to normative ideas. Thereby, the ideas of mutual adjustment “show that some overall pattern of design, which one would have thought had to be produced by an individual’s or group’s successful attempt to realize the pattern, instead was produced and maintained by a process that in no way had the overall pattern or design in mind” (Nozick 1974, p. 18).

Neoclassical economists (Bal and Goyla 1994; Spence and Thomas 1974) have discussed market fashioning using the originally Cartesian notion of economic actors who are like egos or monads, each being self-interested with a set of given preferences, whose activities result, in a natural way, in a market, often with price as the sole means of competition. For many economists the basic assumption of “homo economicus,” the stylized rational utility–maximizing actor is what triggers the development, whereas economic sociologists tend to emphasize identity formation through mutual observation and social structure as the bedrock of emergent markets. Many economists do not theorize the market as such, nor how they come about; the market is more an assumption, and their concern is rather what markets do. Moreover, those who stress mutual adjustment speak about and study markets (or trade in general), not marketplaces. In many cases markets are also considered to be efficient (Fama 1970). The result is that very few economists have written about the *making* of markets.

Friedrich A. Hayek theorized systematically about markets as a specific form of coordination different from organization, by looking at the market process. Hayek claimed that markets grew out of barter, exchange, and trade (von Hayek 1988, pp. 42-43; Plattner 1989, p. 180). The actors involved may even lack a conception of the market, which underlines that it is an “unintended consequence.” Accordingly, markets are “spontaneous orders” (*cosmos*) that evolve out of “chaos,” as opposed to planned orders based on design (*taxis*) (von Hayek 1973, pp. 35-54). While these orders still result from utility-maximizing individuals, Hayek (1945, 1937) stresses the subjectivity of knowledge and the role of prices as central information that allow for a decentralized adjustment of supply and demand beyond any form of “rational” human design.

The notion of mutual adjustment is central to evolutionary economics (Nelson and Winter 1982, pp. 9-10). Evolutionary economists see institutions (rules) as the conceptual cornerstone of their explanations of how markets develop (Hodgson 1996, pp. 175-179). They agree that the rules and institutions of the market emerge because of humans’ impulse to survive (Greif 2006, p. 126; cf. Hannan et al. 2003, p. 309; Hodgson 1996, pp. 170-186). Jean Tirole has in several papers analyzed markets, and not only the theme of mutual adjustment. In one text (Maskin and Tirole 1988), he uses game theory to account for how firms take one another’s behavior into account in oligopolistic markets.

There is another strand of literature, originating in economics, which highlights the role of mutual adjustment, but under conditions of “imperfect” competition (Chamberlin 1933; Greenhut 1975; Robinson 1933). Robinson (1933) thus concludes that firms try to control their marginal revenue by individually adjusting prices through estimates or trial and error, which will eventually lead to a state of “monopoly equilibrium,” rather than following the simple mechanics of supply and demand. Conversely, monopsony describes a market structure in which a single buyer controls demand (Robinson 1933, p. 218); for instance, a large retailer controlling a subcontractor in the automobile industry. Chamberlin (1933) goes even further by dispensing with the assumption of homogenous goods. Thus, while competing with other firms for the same market segment, firms try to gain control over supply and pricing by offering products distinct from those of other producers in the market. More recently, economists have rediscovered the idea of product differentiation and competition by quality niches (Bordalo et al. 2016), though without mentioning those thinkers, like Chamberlin, who have discussed this before. Accordingly, one trigger of new markets and changes in the structure of existing markets is innovation of new products by entrepreneurs, who orient themselves to one another and to the market (Coldwell Daniel 1984, p. 303).

In economic sociology, it is above all Harrison White who has discussed market fashioning using the idea of mutual adjustment. White approached the emergence of what he calls producer markets in terms of the notion of the sociological and relational constitution of actors, or more specifically, of their market identities (White 1992, 2002, pp. 266-283; White 2008). Markets, in his view, evolve historically out of production systems (White 2002). Markets, White says, are “self-reproducing social structures among specific cliques of forms and other actors who evolve roles from observation of each other’s behavior” (White 1981, p. 518). In other words, White’s market definition suggests that he stresses the idea of mutual adjustment (cf. Luhmann 1988). Producers in these producer markets gain identities as a result of how they are viewed by the customers, who in a sense, endow them with status (Podolny 2005). New markets may thus result from differentiation, specialization, and branding when differentiation goes so far that there are several sets of competitors who orient to one another and thereby establish a structure. Others, like Padgett and Powell (2012), support this hypothesis with historical evidence, showing that market formation is the result of stochastic processes over time, rather than the product of human design.

All in all, those who see market fashioning as a mutual adjustment claim that markets evolve from conditions that originally are highly uncertain, or even ambiguous. Once market actors have “a shared frame of perception among its firms” (White 2002, p. 2), we may speak of an ordered market. On the ground, it is the actors’ practices that perpetuate the market: “Whether an institution is explicit or implicit, practices are the vehicles for enacting and reproducing it” (White 2008, p. 173). For a market to come into existence requires basic security and trust; some rights that can be traded; and infrastructure in the form of trading places at which information about the offers and their worth can be observed and where those with an interest in trading can meet. These institutional conditions are not discussed at length by proponents of the approach, or they are simply taken for granted. Another shortcoming is that only a few studies (e.g., Haveman and Nonnemaker 2000) pay attention to actors’ size and market power.

We have seen that those who stress mutual adjustment can explain ordered markets either as a result of rational egos—readymade “economic men”—or as a result of actors who co-evolve and became “rational” while they contribute to the making of markets. Structures in markets are, in either case, fashioned in interaction and direct communication between actors and as a result of signaling and observation. Sociologists in particular stress how order in markets results from actors gaining status in the process of acting as buyers or sellers, indicating that markets do not come about from “nothing.” In reality, markets result from interaction by actors who have status positions prior to their market fashioning activities. However, Hayek acknowledges both the role of the state and attempts by economic actors to organize their efforts. For Hayek, forms of organized market-making may indeed occur, and he finds that also normatively acceptable as long as the state is not in the driving seat of the organization. By this inclusion he broadens the notion of mutual adjustment, and makes it less precise.

## Organization

To analyze market fashioning in terms of organization may at first glance be seen as standing in direct opposition, not only to mutual adjustment, but to the very essence of markets. But as mentioned, the actual market trade—no matter how the market in question came about—must be understood in terms of mutual adjustment of free actors. The concern with this ideal type, however, is with markets that are the result of organizing efforts. Coase (1988) and Williamson (1975) claim that complete (“formal”) organizations, that is, firms—come into existence only when the transaction costs in markets are higher than the cost of setting up organizations (see also Abolafia 1996, pp. 7-8, n. 11, p. 196), though the theme in their texts is not explicitly market fashioning. Some, such as Sombart (1927) see “artificial” (“*künstliche*”) processes, with rules and regulations, merely as deviating from natural market processes in which individual actors trade. Also Polanyi (2001, p. 71) says that “regulation and markets, in effect, grew up together.” To understand how markets were regulated or, as we say here, organized, requires at least a rudimentary understanding of what “organizing” means.

Organized fashioning can be defined as a deliberate attempt to create a market (Ahrne et al. 2015) by making decisions concerning others (Ahrne and Brunsson 2008, p. 49; Luhmann 2000). A decision must be made known and a decision-maker can be held accountable, but it does not follow that it is accepted by everyone. The result of an attempt to organize, or change, a market does not have to be exactly what was intended.

The collective knowledge from the organization literature regarding markets is clearly that markets contains institutions such as regulations, goals, symbols, rites, customs, and conventions (Abolafia 1996, p. 52; Nee and Opper 2015, p. 149; Wherry 2012), but that markets seldom are fashioned through or by means of institutionalization.

In contrast to the fairly clear canon and origin of ideas of mutual adjustment in markets, the sociological literature on market organization is more diffuse and more descriptive. Powell and DiMaggio (1991), for example, talk of organizational *analysis*, not organization *theory.* Some sociologists who have studied the making of markets frequently use organizational elements in their accounts, not least in the performativity literature. The explicit use of organizational analysis, however, is rare. We argue that the theoretical ideas on “organized markets” come from researchers who have observed real markets (e.g., Dezalay and Garth 1996; Scott 2014; Suchman et al. 2001). In other words, the theoretical account is largely generated inductively.

The literature teaches us that the first historical instances of market organization do not concern individual markets but marketplaces. Organizing markets was a sign of power and authority, but also a potential means of procuring incomes. Holding a market was, in a way, constitutive of being a sovereign as it also entailed revenue from taxation (Britnell 1978, p. 189; Skre 2007, p. 453; Weber 1978, p. 114; Weber 1981; Weber 1998, p. 163).

The first formal exchanges, which in our language means that we are talking of market places, in which physical goods were traded were set up in Bruges (1409) and Antwerp (1460) (Markham 2002, p. 94). A qualitative step was taken when the goods traded no longer had to be physically brought to the stock exchanges. Thus, to avoid the cumbersome physical movement of objects, as well as physical money, trade turned into an exchange of rights or claims. Weber (1981, 1999) has described this development and also spelled out the practices of the different exchanges. Other sociologists have repeated this point (Merrill and Palyi 1938). In a volume edited by Polanyi, Arensberg and Pearson (1957), several examples of organized marketplaces are discussed, supporting the idea that markets were organized. Information on how these markets came about, however, is limited.

The marketplace in Carpentras, we learn, is organized with the help of a set of rules decided by the city authority (Pradelle 2006). The behavior of stallholders is monitored by the city authorities. The actual commerce—the trading—is not governed by the public authorities, but decided by market actors, sellers and buyers. All of this is embedded in the culture of this market. There are other examples of how market places have been set up, for example, Roman bazaars (Bang 2008, pp. 239-289), as well as other contemporary physical market places (Black 2012; Dewey 2020).

A stock exchange is the paradigmatic example of an organized market. Two of the most prominent researchers—who are also seen as founding fathers of the neoclassical model of the perfect market—Alfred Marshall and Leon Walras, both grounded their theories in the observation of real stock exchanges, in London and Paris. Alfred Marshall is clear about the central role organizations play in fashioning exchanges: “The most highly organized exchange is the Stock Exchange. As a rule those who deal in it are in effect a corporation: they elect new members, as well as the executives of their body and appoint the committee by which their own regulations are enforced” (Marshall 1920, pp. 256-257). Walras has a similar view: “The markets which are best organized from the competitive standpoint are those in which purchasers and sales are made by auction…. This is the way business is done in the stock exchange, commercial markets, grain markets, fish markets, etc.” (Walras 1954, pp. 83-84).

Carl Menger, albeit in relatively vague terms, claims that “organized markets,” beginning with markets for certain goods, are the condition for having prices as long-distance signals (1976, pp. 248-250). By extensive and carefully designed organization, many contemporary stock exchanges adhere to the image of the "perfectly competitive" or so-called ideal market (Knight 1921, pp. 76-81). This means that the objects are homogenous, and that trade can be done in a standardized way.

There is also plenty of empirical research on both historical and contemporary organization of marketplaces and markets. Although the initial stages of what were later to become financial markets in London are perhaps best described in terms of mutual adjustment, many of the later ones were molded by legislators’ decisions to organize such markets. London stock market was already “active and organized” by 1712, according to Carruthers (1994, p. 170). Murphy (2009, p. 83) describes in detail how, for example, steps were taken to limit the number of brokers as early as 1773, when the body of the London Stock Exchange was funded (Smith 1929). Many marketplaces were mapped on each other. The Chicago Stock Exchange, for example, is mapped on the New York Stock Exchange (Merrill and Palyi 1938, p. 561, n. 1). More generally, Marshall states that “stock exchanges … are the pattern on which markets have been and are being formed for dealing with many kinds of produce which can be easily and exactly described, are portable and in general demand” (Marshall 1961, p. 328). We thus find a considerable body of work on the organization of financial marketplaces (Braudel 1992; Carruthers 1996; Cope 1978; MacKenzie and Millo 2003; Murphy 2009; Preda 2009; Smith 1929). In addition, anthropologists (Arnold 1957; Geertz 1963; Geertz 1992), geographers (cf. Power and Jansson 2008) and of course historians (Braudel 1992) all point towards the same thing, namely that marketplaces are organized. There are many examples in the literature of marketplaces and how individual markets operate “within” a marketplace.

Aspects of market organization are also prominent in accounts underscoring the role of knowledge regimes and non-market actors, such as economists, as key to the understanding of market-making (Garcia-Parpet 2007). This literature, following Callon (1998), that sits under the umbrella of performativity essentially views markets as organized. Callon clearly sees this: “the market implies an organization, so that one has to talk of an organized market (and the possible multiplicity of forms of organization) in order to take into account the variety of calculative agencies and their distribution” (Callon 1998, p. 3). The making of carbon emission righst markets (MacKenzie 2009; Rosenström 2014), and electricity markets (Breslau 2011) are additional examples of organization of markets. Kirman concludes that “each market [of those he studied] is characterized by an organization and a structure that have an impact on the observed outcomes (Kirman 2001, p. 191). The same can be said about the organization of large wholesale markets, such as Tsukiji in Japan (Bestor 2004, pp. 177-204) and the organization of new electronic auction markets in which commodities, buyers, and sellers are spatially dispersed (Dobeson 2016). In addition, the growing number of electronic platforms give us plenty of examples of electronic marketplaces (Walia 2013). Regulation is a form of fashioning of markets. Almeling has analyzed the regulation of the US market for human eggs and sperm (Almeling 2007), which previously was a non-good (cf. Zelizer 1981) and Levy Spiller the regulation of the electricity industry (Levy and Spiller 1994). The state can also fashion markets, such as the lottery market, made by regulations to increase tax revenues (Beckert and Lutter 2009). Regulation of labor markets is another clear example of how markets are at least partly organized (Botero et al. 2004). Other economists (Laffont and Tirole 1990) have studied regulation of firms with monopoly positions in markets. Moreover, also de-regulation, for example, in the form of privatization of activities that lead to markets (Brunsson and Jutterström 2018), is an example of how decisions lead to markets. Although the state is important, it is not necessary as an organizer of markets. States may also trigger self-regulated markets, for example, the setting of industrial standards (Gupta and Lad 1983; Mollgaard 1997), or markets that are self-organized markets, to avoid the threat of regulation by the state. Attempts to organize markets using regulations do not, of course, necessarily succeed. Thiemann and Lepoutre (2017) show how evasion of rules by market actors shapes the way the market is fashioned, and attempts to control markets by the state can lead to the creation of black markets (Healy 2006, pp. 123-127). Various forms of illegal markets (Ruggiero and South 1997) exemplify how markets are organized without the help of the state.

We can say that exchanges are organized marketplaces in which a great number of different listed markets, for the different stocks or for other standardized or homogenous goods, are embedded. It is also clear that there are many actors, among the states, who by their attempts to organize markets fashion the markets in the real economy. Those who discuss organized fashioning suggest that what the market is about—how to do things in the market, its institutions, perhaps even its culture (cf. Zelizer 1979), and how prices are determined—essentially has to be addressed, though not necessarily decided, prior to the “launch” of the market. In addition, other market institutions, such as useful devices for measuring quality, must be known by market actors, so that they can be employed to fashion markets. This also assumes that there are actors who have a reasonably clear idea of who they are and what they want, what the market is (if it is about to be changed) or will be about (if it is a new market), and how it will work. In practice, organized market fashioning presumes knowledge among actors in these situations about market institutions, which means that the level of ambiguity must be considerably less for the fashioning to get started than what is necessary for market fashioning by means of mutual adjustment. We now turn to the final section that sees market fashioning as a political power struggle that is settled in fields.

## Fields

If mutual adjustment approaches focus on actors’ interest and how their actions unintentionally fashion markets, and organizational approaches zoom in on the decisions that actively fashion markets, the ideal type of fields, reflected in the works of Bourdieu and Fligstein, presumes that the context and conditions of market fashioning should be at the center of our analysis. Thereby attention is directed towards social conditions, the positions of actors and organizations and their power and interests that enable and restrict the decisions of individuals of all types, including attempts to organize markets. As Jens Beckert puts it: “By understanding markets as fields, we shift the emphasis in the analysis of markets from the act of exchange to these structuring forces” (Beckert 2010, p. 609). This shift, also, means that the focus is no longer on the core of the market and its construction, but rather on the conditions that may further or constrain the institutions of new markets. Put differently, though we claim that fields are of great relevance for understanding the context and conditions of market fashioning, this ideal type does not primarily refer to the origin of markets.

Fields represent a way of analyzing the conditions and forces needed to understand market fashioning, in addition to other attempts to account for “context,” such as social worlds (cf. Becker 1982). The notion of field is used to create an image of both open fields and magnetic fields. It is primarily the latter, drawing on the notion of “power,” that has been the source of inspiration for those who study the social in terms of fields in the social sciences. By using the concept of field, the overlapping, “nested,” “embedded,” or “Russian doll” logic of fields within fields (Bourdieu 1996, pp. 114ff.; Bourdieu 2005, pp. 89ff.; Fligstein and McAdam 2012, pp. 9, 80) is put at the center. Key notions of field theory include power, positions (in relative terms), struggle over positions and “stakes” in the field, strategies of “conservation” and “subversion” tied to positions (Lahire 2014, p. 66), perceptions of the field—which is captured by Fligstein’s notion of “conception of control”—as well as field boundaries. The field literature on market fashioning refers to both mutually adjusting actors and organization.

Bourdieu makes use of markets in several texts, albeit often metaphorically, such as “the linguistic market” (Bourdieu 1977), or the “marriage market” (Bourdieu 1977, pp. 54ff.). In only a few instances (Bourdieu 1996; Bourdieu 2005) is Bourdieu concerned with markets. Most notable are his studies of, and how, fields of production and the field of consumption are constructed.

According to Bourdieu, each field, in fact, represents only one side of the market, and Bourdieu suggests that meetings between actors in two separate fields (for example, a field of consumers and producers) happen due to “the homology between the space of producer and the space of consumers” (Bourdieu 1996, p. 249). Seen in this way, each “half” of a market is a field in its own right, but each market is also situated in relation to other fields, adjacent and also “embedded” in larger fields. “Homology” between actors situated in two distinct fields (i.e., what we label as a market) is not a mystery of matching, but rather an obvious coming together of actors with a similar habitus.

A market can be said to come into existence when enough actors, struggling with one another over certain forms of capital, gain enough autonomy, if this struggle is not determined by outcomes in other fields. In an emerging field “anything goes.” Actions may be aiming at a market or not, and the result may be a market or not. Thus, both mutual adjustment and organization may be relevant. What is striking, especially in the early phases of the emergence of a field, is that it gets much of its “logic” from adjacent fields (Bourdieu 1996).

To control a market (field), actors may use “power tactics” (Santos and Eisenhardt 2009, p. 648). But power is unevenly distributed; the state is a powerful controller of many, if not all, legal markets. Bourdieu gives several examples, but says “there are, no doubt, few markets that are not only so controlled as the housing market is by the state, but indeed so truly constructed by the state …” (Bourdieu 2005, p. 89). However, even though the state can be a market actor (for example, buyer or seller) and clearly is *the* market fashioner according to Bourdieu, individual actors can play a role in fashioning: “To impose a new producer, a new product and a new system of taste on the market at a given moment means to relegate to the past a whole set of producers, products and systems of taste, all hierarchized in relation to their degree of legitimacy” (Bourdieu 1996, p. 160). Even a relatively small change in an existing market has to be accompanied by a whole change in the “institution of the market.” As part of the market, not only formal institutions, but also actors’ tastes, perceptions, and positions have to be altered. The advantage that power gives actors is great, but it is also clear that players with relatively little power can change the market by their activities at a certain time with the right habitus. The overall picture that appears after having read Bourdieu is that markets are fashioned from existing fields, not created or invented out of thin air. Powerful players may use existing rules and strategies or to some extent import them from adjacent fields, and the market can be fashioned from within or from outside—that is, from fields—some of which may be markets. Bourdieu’s somewhat metaphorical use of markets has rarely been used by social scientists for analyzing real markets empirically, not even among his followers. We do not see his approach as particularly well suited to this either, primarily because of its metaphorical usage and its lack of precise concepts that refer to empirical markets and market fashioning.

DiMaggio and Powell (1983) address the question of homogeneity of organizations and assert that a set of firms that make up a market, for example, operate in one and the same field. By organizational field they mean the organizations that “in aggregate, constitute a recognized area of institutional life: key suppliers, resource and product consumers, regulatory agencies, and other organizations that produce similar services or products” (DiMaggio and Powell 1983, p. 148). Thus, not only buyers and sellers are included in the field, but a set of actors, which portrays the field as an “industry.” Studies have also shown that the structure of the market affects the number of entrances (Haveman 1993). DiMaggio and Powell (1983) argue that the order of a field, observed in terms of similarity of organizations and their practices, emerges over time as a result of “a diverse set of organizations” (ibid.). In contrast to Bourdieu’s thick description of institutionalization as equal to, or determined by socialization, DiMaggio (1988) sees actors’ interests as separable from the institutions they uphold in and by their practices (Friedland and Alford 1991, pp. 244-252). Thus actors are, according to DiMaggio, given both the desire and possibility to be “institutional entrepreneurs” that fashion new markets. Scott's (2014, p. 117) distinction between “organizational and institutional entrepreneurs” is an attempt to qualify statements such as DiMaggio’s, and potentially to bridge the thick and thin depictions of institutionalization perspectives. Organizational entrepreneurs are people who try to *change* an existing market within an existing institutional mold, while institutional entrepreneurs have an interest in fashioning a whole new institutional setting. The depiction of institutionalization is clearly as thick as Bourdieu’s, but it does open up for a more agency- or actor-based outlook on fashioning, that also is found—and yet somewhat altered—in the field theory discussed below.

Neil Fligstein, after White, is the sociologist who has had the biggest impact on sociological research on markets and market fashioning. Fligstein’s “markets as fields” (Fligstein 1990, 2005, pp. 192-194) states that “markets depend on extensive social infrastructure … stable states and currency, the rule of law, functioning property rights, governance structure, and rules of exchange” (Fligstein 2005, p. 194). The concrete markets as discussed by, for example White, however, are not the focus of the analysis. The recipe for making markets, in short, is to have “participants (i.e. firms), rules, and usually governments to create and enforce rules” (Fligstein 2005, p. 194). A more recent text that Fligstein wrote with McAdam stresses that to create a market/field at least two participants are needed, and “participants” can here be groups and/or social movements, as well as firms (Fligstein and McAdam 2012, p. 86). Fligstein draws on White’s market definition, but emphasizes collaboration between competitors as an important tactic to survive in the market (Fligstein 2001a, p. 7). Markets can thus be understood in terms of “politics” and hence “fields”; in other words, they can be seen as arenas for power struggles but also coalitions (Fligstein and Mara-Drita 1996; Fligstein 2001a, p. 12; Hellman 2007).

For a new field, such as a market, to come into being four issues need to be settled:

1. “What is at stake”—actors naturally have differing interest and opinions, as well as differing amounts at stake in the emerging market, but there needs to be “consensus,” at least about what the market is about.
2. A relatively stable or fixed number of actors with (socially) acknowledged positions.
3. A shared understanding of the rules of the game, for example, intersubjectively acceptable behavior in pursuit of interests.
4. A “broad interpretive frame that individual and collective strategic actors bring to make sense of what others within the strategic field are doing” (Fligstein and McAdam 2012, pp. 88-89).

In light of the settlement of these four issues, Fligstein and McAdam (2012, pp. 149ff.) analyze how market fashioning has similarities to the birth and evolution of social movements. Their work takes the historical conditions under which fields and markets come about seriously, and claim that

one can conceive of emerging fields as social spaces where rules do not yet exist but where actors, by virtue of emerging, dependent interests and worldviews are being forced increasingly to take one another into accounts in their actions.… So, for example, “new” product markets are often founded near “old” product markets as part and parcel of the search to achieve stability for the firms. (Fligstein 2001a, p. 87; cf. Fligstein and McAdam 2012)

In other words, new markets result from different firms watching one another, to mirror White. In contrast to White's theory of producer markets, and more in line with both Becker’s “world” concept and DiMaggio’s notion of “industry,” the strategic action field approach embraces non-commercial actors situated "outside of the market." In fact, new markets can spring from grassroots movements, as McInery (2014) has shown in his study of the emergence of a new market for IT services. In a similar vein, Weber et al. (2008) have shown how the movement for grass-fed meat and dairy products has challenged conventional production standards and practices in order to establish a new market niche by mobilizing cultural codes of authenticity and sustainability. Dobbin sees the economy and its markets as reflections of the polity Dobbin 2011).

All in all, some field definitions claim that markets are “just” fields, but the literature also accounts for what is happening outside the specific market in question. In our view, the most fruitful is to see markets as being *in* fields. The strength of the works that study markets in terms of fields is that markets are connected with their environment, which is largely missing from the other two ideal types we have analyzed.

Two shortcomings of the theoretical notion of field hinder the works drawing on fields from giving a full account of how markets are fashioned. The first is that they do not mention the variety of fashioning that we can observe, reflected in the conceptualization of ideal types of mutual adjustment and organization. This also means that a detailed analysis of how the market is fashioned, in terms of how prices are determined at the level of concrete markets, is rarely done. The second shortcoming refers to a peculiar feature that makes field different from how markets are presented by mutual adjustment and organizational scholars, namely that it divides the market into two halves, or two fields: “producers/sellers” and “consumers/buyers.” This can make it unclear not only who is in a market, but also who can fashion it. More importantly, it leaves the question of markets hanging. Bourdieu defines those who are in a field as persons who have “stakes in the game” (Bourdieu and Wacquant 2002). Bourdieu, however, has little interest in identifying “institutional entrepreneurs” (DiMaggio 1988; Scott 2004), that is, the skillful actors that Fligstein and McAdam (2012, pp. 109ff.) view as central to explaining the emergence of fields/markets (also see Fligstein 2001b; Stark 2009). Fligstein and McAdam refer to fields as embedded in other fields, but only those who “*routinely* take each other into account in their actions” (2012, pp. 167-168) are members in a field. Consequently, by this way of reasoning, the producers, but not the consumers, constitute a field (Fligstein and McAdam 2012, p. 168). Moreover, the clearly Whitean approach to markets, with actors who share a role are watching one another, gives Fligstein’s approach an unfortunate “bias” towards mutual adjustment.

## Discussion

Market fashioning refers to how new markets come about. Mutual adjustment and organization represent two contrasting ideal types of market fashioning, which cannot be reduced to one another. To view fashioning in terms of mutual adjustment demonstrates how markets, essentially unintentionally, can result from interaction of actors. Marketplaces can probably be changed as a result of mutual adjustment but are unlikely to be created in this process. The organizational ideal type demonstrates that markets and marketplaces can intentionally be changed or be made by actors’ decisions.

The third ideal type, fields, offers much on the context of actors and institutions involved in market fashioning processes. The idea of field implies that markets do not come from nowhere. Actions that deliberately or unintentionally lead to the fashioning of markets or marketplaces gain agency, for example, from the positions of the actors in the field, from their status, identities, entrepreneurial skills, and the fact that they can use the available institutions and cultural scripts. While offering some ideas of the context, it is at the same time silent about what types of markets will emerge, though some Whitean ideas are implicitly imported. It is, strictly speaking, not a theory of the origin of markets. We have also noted that many field-oriented studies are weak on the actual market: what is traded, how it is done, and how the value and prices of the objects traded are determined. Bourdieu and Fligstein have taken examples mainly from markets in which producers are differentiated, but we claim that notion of field is not inherently tied to a specific type of market. If the distinction between mutual adjustment and organization is assumed, and their respective ideas on how concrete markets are fashioned are employed, the notion of field informs us about the conditions of market fashioning, but less on the process or the market outcome. To address the more concrete process of how markets are fashioned, we must employ either mutual adjustment or organization. The scope conditions of field theories are thus larger, at the same time as the approach is less distinct.

Table 1 presents a brief meta-analysis of our discussion by identifying what characterizes each of the ideal types, as well as mentioning their strength and weaknesses. Based on this, we argue that the two different ideal types of market-fashioning—mutual adjustment and organizing—are related to specific markets. This meta-analysis gives some direction for additional research to find out of how different forms of markets appear to be fashioned.

**Table 1:** Key features, strengths, and weaknesses of the three market fashioning ideal types identified: mutual adjustment, organization, and fields.

Ideal types of market fashioning			
	Key features	Strengths	Weaknesses
Mutual adjustment	Markets as unintended consequences of actions that aim for trade and relational positioning of different actors.	Explains producer markets characterized by status order in which actors are identified as buyer or seller. Typical example: producer markets with differentiation and quality uncertainty, such as fashion markets.	Overemphasis on individual actors and lack of market making context. Cannot explain exchange markets with standardized objects of trade. Typical example: stock exchanges. Cannot explain fashioning of marketplaces.
Organization	Markets as a result of intentional decision-making for others.	The role of planning, and explicit decision-making for the fashioning of markets. Explains exchange markets with standardized objects of trade. Typical example: stock exchanges. Explains marketplaces.	Overemphasis on intentional decision-making; somewhat lacking market-making context. Cannot explain producer markets characterized by status order in which actors are identified as buyer or seller.
Fields	Markets as dynamic social structures in which incumbent firms and challengers struggle to maintain and establish market power.	Structural change as a result of dynamic power struggles between economic and non-economic fields, for example, social movements and established market structures. Market-making context.	Fuzzy conception of boundaries between fields. Unclear on the market/marketplaces distinction. No coherent idea of what forms of markets fields fashion.

Our analysis suggests that mutual adjustment gives a good account of the markets characterized by differentiation of products. These markets lack an institutionalized and objective standard for evaluating quality, independently of who is selling or buying; instead status becomes the ordering principle. It is here suggested that these markets emerge unintentionally as a result of individual actions and are ordered according to status of those trading; some sellers have more status than others. In other words, status cannot be the consequence of decisions; it is a result of interaction and status is given and acknowledged (e.g., Mears 2011; Menger 2014; Podolny 1993). In these markets ordered by status, product differentiation and identities of sellers and buyers are characteristic traits. Most consumer markets are of this kind, such as fashion markets, art markets (Aspers 2010; Beckert and Rössel 2004; Velthuis 2005), wine markets (Garcia-Parpet 2011) but also many producer markets, for example, the classic example of a market for frozen pizzas (White and Eccles 1987) is ordered according to status.

The ideal type of organization is associated with intentional attempts at making and fashioning marketplaces, as well as one typical market form, exchanges of standardized and homogenous products (stock exchanges are the ideal example). When it comes to exchanges, for example, of stocks or many agricultural products, the goods are standardized, and, in principle, it does not matter whom one is trading with. In contrast to status, which cannot be “decided,” standards (Aspers 2009) can be decided by market actors or actors who are not involved in the direct trade of the market, such as the state. Standards help to order markets. In markets ordered by standards actors seek out trading partners not because of their status, but because of the ease of adjudicating differences between offers using one, or sometimes, several standards. Examples of standards are objective quality measurements to adjudicate between offers, or when the offers are completely standardized (homogenous), as in the neoclassical model, based on just one dimension, price. Offers in standard markets can be objectively determined, independently of the actors present in the market.

The identified affinity with the ideal types of market fashioning, and the forms of markets, point at the possible to dissolve a set of preconceptions that currently obfuscate progress in market studies. We have seen that the perfect market, which is the benchmark and often the assumption in economics, in fact requires much organization, but many economists take perfect markets for granted; “in the beginning there were markets, ” as famously stated by Williamson (1975, p. 20). Many neoclassic economists seem to view perfect markets as emerging naturally, thereby neglecting the processes of market fashioning. More generally, since in economics “the discussion of the market itself has entirely disappeared” (Coase 1988, p. 7), market fashioning is unlikely to be given more attention. What we see, though, is a major discussion in economics of changes in markets, for example, by means of regulation. Quite a few sociologists, as mentioned above, have studied the organization of these “perfect markets,” for example, Donald Mackenzie (MacKenzie and Millo 2003; MacKenzie 2009).

The majority of markets, as noted above, are not perfect markets. In his influential textbook *Economics,* Samuelson argues that pure competition exists only in a few agricultural industries, in the form of organized exchanges and auctions, whereas imperfect competition exists in most industries, in markets characterized by rivalry, as explained by Chamberlin (Cramer and Heuser 1960, pp. 395-396; Varian 1987). The fashioning of these more frequently existing markets, we argue, has rarely been subject to study by economists. However, more recent trends in economics, such as game theory and behavioral economics, as well as institutional economics, offer more nuanced views on market fashioning.

To understand these producer markets with non-homogenous commodities, in contrast, has been a large topic in economic sociology. The problem here is that very few sociologists have embraced the ideas of mutual adjustment. This is somewhat surprising since Harrison White, who triggered the discussion in new economic sociology, has built his thinking on mutual adjustment. Many sociologists have stressed organization, field, and embeddedness, despite the fact that the markets they have studied to a large extent are suitable for the concept of mutual adjustment.

## Conclusion

We propose that, to overcome the conundrum of market form and market fashioning processes, one should not speak of one theory that is correct for all markets. Instead, we suggest that different market forms and research questions require different theories to account for how they are fashioned, or use the different theories to account for one case over time as does Spicer (2012). We think this can help to dissolve some of the current debates on the right and wrong theories of markets. Our finding implies that the economic notion of market—the perfect market—has empirical counterparts in contrast to what many sociologists assume who frequently criticize markets, economic man, and neoclassical market theory. It also suggests that the view that at least some economists seem to hold—that there is only one form of market—is inaccurate. To generalize: both economists and sociologists of market studies have been right and wrong in our view. However, the reasons for this ambivalence, we believe, have not been clear. Sociologists should perhaps be more explicit about the role of mutual adjustment. Economists should, in contrast, pay more attention to fields and above all organization of markets, a theme that was evident to classical economists, but which was more or less lost with the institutionalization of neoclassical economics.

In this text we do not propose a coherent theory of market fashioning. What we have done is to identify ideal types of market fashioning. We have also identified some affinities between these ideal types and the market forms. To move from affinity to explanations requires causality. To address the causal processes in detail requires that we trace market fashioning over time, which obviously put some restrictions on researchers. Another central theme to study in the future would be the potential of a more all-encompassing market fashioning theory. As it is now, the three ideal types are not integrated, nor is their relation clear. However, at least some historical works (Bühler 2019; Engel 2019; Jeggle 2009) that are theoretically informed, point to how the ideal types may be combined in reality and understood in relation to one another over time or in different combinations. Moreover, comparative case studies of different types of markets and their social expectations open new avenues for theorizing market fashioning (Dobeson and Kohl 2020). We suggest that these theoretical ideas could guide future empirical research to pursue empirical work to understand contemporary as well as historical market fashioning.
